# Investigating interindividual variability in corticomotor reorganization during sustained hamstring pain: A randomized experimental study

**DOI:** 10.1002/brb3.2996

**Published:** 2023-04-11

**Authors:** Rocco Cavaleri, Jawwad Imam, Ebonie Rio, Nadia Moukhaiber, Daniel Thomson, Ariane Suhood, Simon J. Summers

**Affiliations:** ^1^ Brain Stimulation and Rehabilitation (BrainStAR) Lab, School of Health Sciences Western Sydney University Sydney New South Wales Australia; ^2^ School of Biomedical Sciences Queensland University of Technology Brisbane Queensland Australia; ^3^ School of Allied Health La Trobe University Melbourne Victoria Australia

**Keywords:** Hamstring, TMS, Pain, Corticomotor

## Abstract

**Background:**

Increasing evidence suggests that pain drives maladaptive corticomotor changes that may increase susceptibility to injury and promote symptom recurrence. However, few studies have evaluated the influence of interindividual corticomotor responses to musculoskeletal pain. Existing research in this area has also been limited largely to the upper limb. This is a pertinent point, given the functional and neurophysiological differences between upper and lower limb muscles, as well as the fact that most acute sporting injuries occur in the lower limb. Accordingly, this study explored the variability of corticomotor responses to experimentally‐induced sustained hamstring pain and whether specific patterns of corticomotor reorganization were associated with poorer outcomes (mechanical sensitivity, pain, or functional limitation).

**Method:**

Thirty‐six healthy individuals participated. Following random allocation on Day 0, the experimental group performed an eccentric exercise protocol of the right hamstring muscles to induce delayed onset muscle soreness. The control group performed repetition‐matched concentric exercise that did not induce soreness. Measures of mechanical sensitivity, pain, function, and corticomotor organization were collected at baseline and on Day 2.

**Results and conclusions:**

Corticomotor responses to sustained hamstring pain were variable. Individuals who developed corticomotor facilitation in response to hamstring pain experienced greater mechanical sensitivity than those who developed corticomotor depression. These novel data could have implications for rehabilitation following lower limb pain or injury.

## Graphical abstract

This was the first study to explore the variability of corticomotor responses to sustained lower limb pain. Corticomotor responses varied considerably between participants. Individuals responding with corticomotor facilitation experienced increased mechanical sensitivity compared to those responding with corticomotor depression. Our data raise the possibility that corticomotor facilitation in response to lower limb pain could indicate susceptibility to greater mechanical sensitivity and delayed return‐to‐play following injury.

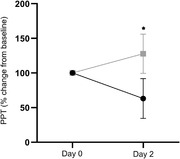

## 1 INTRODUCTION

Accumulating evidence suggests that pain from musculoskeletal injuries drives maladaptive corticomotor changes that may promote symptom recurrence (Schabrun et al., 2015; Tsao et al., 2008, 2011). Through the use of transcranial magnetic stimulation (TMS), several studies have identified changes in corticomotor activity in response to pain, characterized by shifts in the cortical representations of affected muscles (Schabrun et al., 2017; Te et al., 2017) and changes in corticomotor excitability (Mhalla et al., 2010; Salerno et al., 2000; Strutton et al., 2005; Te et al., 2017). These corticomotor changes have been associated with pain intensity and functional limitation (Cavaleri et al., 2021; Seminowicz et al., 2019; Tsao et al., 2008). Maladaptive changes in corticomotor pathways are hypothesized to contribute toward injury recurrence by promoting abnormal movement patterns that detrimentally alter soft tissue loading (Hodges & Tucker, 2011). Observations of neuromuscular inhibition and deficits in proprioceptive processing following musculoskeletal injury support potential cortical adaptations to acute and chronic pain (Buhmann et al., 2022; Fyfe et al., 2013; Summers et al., 2021).

Specific patterns of pain‐induced corticomotor reorganization may be associated with poorer outcomes over time. Indeed, a recent review demonstrated that individuals displaying reduced corticomotor excitability in response to acute experimental pain (lasting minutes) report lower pain severity than those showing increased excitability (Chowdhury et al., 2022). However, reduced excitability was associated with greater pain severity in individuals with *sustained* pain (lasting days‐to‐weeks), suggesting that persistence of this adaptation may be implicated in symptom chronicity or recurrence (Chowdhury et al., 2022). Such data suggest that maladaptive corticomotor reorganization may be a novel risk factor that could be targeted to attenuate pain and reduce risk of reinjury.

While valuable, the exploration of corticomotor responses to musculoskeletal pain has been limited primarily to the upper limb (Chowdhury et al., 2022). This is a pertinent point, given that up to 80% of acute sporting injuries occur in the lower limb (Murphy et al., 2003). Evaluation of lower limb corticomotor responses to acute pain is also essential given the functional and neurophysiological differences between upper and lower limb musculature (Kesar et al., 2018). Hand representations, responsible for fine motor control, are larger, more excitable, and have greater connectivity with structures promoting endogenous opioid release than proximal and lower limb representations, which drive gross motor functions (Andre‐Obadia et al., 2018; Palmer & Ashby, 1992). The capacity for corticomotor reorganization may therefore differ between these regions. Indeed, region‐specific differences in corticomotor responses are supported by work demonstrating that reorganization following upper limb training is graded from proximal to distal, with hand representations showing greater reorganization than shoulder representations (Krutky & Perreault, 2007).

Here, we explored the variability of corticomotor responses to experimentally‐induced sustained pain and whether specific patterns of reorganization were associated with poorer outcomes (increased mechanical sensitivity, pain, or functional limitation). Pain was induced in the hamstring muscle as this region is one of the most commonly injured in the lower limb, and hamstring strains are associated with a high recurrence rate (Brooks et al., 2006; Hawkins et al., 2001; Malliaropoulos et al., 2018; Orchard & Seward, 2002). We hypothesized that sustained hamstring pain would elicit variable patterns of corticomotor reorganization, with increases in corticomotor excitability being associated with poorer outcomes.

## 2 METHOD

### 2.1 Participants

Thirty‐six healthy participants (20 female, 16 male) were recruited for this exploratory randomized controlled study. Participants were recruited through advertisements on Western Sydney University notice boards, social media pages, and the university Physiotherapy program website. A snowball approach was then utilized to broaden the sample and identify additional volunteers.

Individuals were eligible for inclusion if they were aged greater than 18 years and were right‐foot dominant as determined by the Waterloo Footedness Questionnaire (van Melick et al., 2017). Participants were excluded if they presented with a history of a hamstring injury, other major lower limb injuries or surgeries, contraindications to exercise as determined through the Physical Activity Readiness Questionnaire (Helmerhorst et al., 2012), or contraindications to TMS as determined through the TMS Adult Safety Screen questionnaire (Rossi et al., 2011, 2020).

All participants received written and verbal descriptions of the experiment and provided written informed consent prior to testing. All procedures were approved by the local institutional Human Research Ethics Committee (H14252) and performed in accordance with the Declaration of Helsinki.

### 2.2 Experimental protocol

Participants attended two experimental sessions (Day 0 and 2). Participants were requested to refrain from exercise 48 h prior to testing and throughout the duration of the study. On Day 0, demographic (age, sex, height, and weight) and affective‐emotional (Depression, Anxiety, and Stress Scale [DASS‐21]; Osman et al., 2012) data were collected. Baseline mechanical sensitivity (pressure pain thresholds [PPTs]), pain (area, intensity, muscle soreness), functional (lower extremity functional scale [LEFS] (Binkley et al., 1999), single‐leg hop test, maximal voluntary isometric contraction [MVIC]), and corticomotor organization (TMS maps) outcomes were also collected.

Randomization to either an eccentric (pain‐provoking) (*n* = 26) or concentric (non‐pain‐provoking) (*n* = 10) exercise protocol was performed by an independent assessor using a random number generator before testing on Day 0. A larger sample was recruited for the eccentric group to enable sufficient exploration of interindividual variability in response to sustained hamstring pain. Allocation was concealed using consecutively numbered opaque envelopes. Participants completed their allocated exercise program at the end of the session on Day 0. The participants were requested to refrain from treating their pain until the outcome measures were reassessed on Day 2. This included refraining from administering any form of analgesics, lower limb exercise, or using adjunct treatment modalities for pain such as heat, ice, or massage.

### 2.3 Experimentally‐induced pain protocol

#### 2.3.1 Experimental group: eccentric (pain‐inducing) exercise protocol

Twenty‐six individuals were included in the experimental (pain‐inducing eccentric exercise) group. The eccentric protocol was conducted using a Biodex isokinetic dynamometer (Biodex Multi‐Joint System 3, Shirley, NY, USA). Participants were seated upright and secured to the seat using straps over the shoulders and their lap, with their hips and knees in 90° of flexion. The rotational axis of the dynamometer was aligned with the lateral femoral condyle of the right leg. Immediately following a warm‐up (3 sets of 10 eccentric hamstring contractions, with each set gradually decreasing in velocity [90, 75, and 60°/s, respectively]), participants completed 10 sets of 10 maximal eccentric contractions of the right hamstring muscles through a 90° knee flexion range of motion and an angular velocity of 45°/s. The participants were instructed to resist maximally as the lever arm pushed their leg into knee extension. As the lever arm returned back to the start position (0°–90°), the participants were asked to relax and allow their leg to passively return to the starting position (Johansson et al., 2007). Participants rested for 30 s after each set. Standardized verbal encouragement was provided by the investigators. Previous research has shown that such protocols induce delayed onset muscle soreness (DOMS) that develops within 24 h and peaks between 48 and 72 h postexercise (Armstrong, 1984). Following each set, participants quantified their exertion levels using a rating of perceived exertion (RPE) scale from 0 (no exertion) to 10 (maximal exertion).

#### 2.3.2 Control group: concentric (pain‐free) exercise protocol

Ten individuals were included in the control (pain‐free concentric exercise) group. Participants were seated upright on the dynamometer and secured with their hips in 90° of flexion and knees in 0° of flexion. Immediately following a warm‐up (3 sets of 10 concentric hamstring contractions, with each set gradually decreasing in velocity [90, 75, and 60°/s, respectively]), participants completed 10 sets of 10 maximal concentric contractions of the right hamstring muscles through a 90° knee flexion range of motion and an angular velocity of 45°/s. The participants were instructed to maximally bring their knee into flexion against the lever arm. As the lever arm returned back to the start position, the participants were asked to relax and passively allow the knee to straighten (Johansson et al., 2007). A concentric protocol accounts for the effort and fatigue experienced by the experimental (pain) group, without inducing DOMS or changes in mechanical sensitivity (Armstrong, 1984; Pérez et al., 2023). Participants rested for 30 s after each set. Following each set, participants quantified their exertion levels using a RPE scale from 0 (no exertion) to 10 (maximal exertion).

### 2.4 Assessments

All mechanical sensitivity, pain, and functional outcomes were collected by the same investigator, as were all TMS‐derived corticomotor maps. All assessors were blinded to group allocation.

#### 2.4.1 Mechanical sensitivity

##### 2.4.1.1 Pressure pain threshold

PPT was assessed over the biceps femoris muscle belly, located halfway between the ischial tuberosity and the lateral epicondyle of the tibia (Hermens et al., 2000). The location of the muscle belly was confirmed via palpation by an experienced physiotherapist. Force was applied perpendicularly to the skin using a handheld algometer (Somedic, 1 cm^2^ probe) and gradually increased at a rate of 30 kPA/s. The PPT was defined as the point at which the sensation of pressure was first reported to become a sensation of pain (De Martino et al., 2018; Rocha et al., 2012). Three measures were taken and the mean PPT recorded. This measure has been shown to have excellent test–retest reliability (ICC 0.94) (Balaguier et al., 2016; Jones et al., 2007).

### 2.5 Pain and muscle soreness

#### 2.5.1 Pain intensity

Participants scored their hamstring pain on an 11‐point numerical rating scale (where 0 = no pain, and 10 = the worst pain imaginable) at rest, as well as reaching for the floor with knees straight and during the hamstring‐drag test (Zeren & Oztekin, 2006). The hamstring‐drag (or “taking off the shoe”) test is a provocative assessment with a high sensitivity and specificity for biceps femoris strain (Zeren & Oztekin, 2006).

#### 2.5.2 Muscle soreness

Muscle soreness was assessed using a modified 7‐point Likert scale with the following categories: 0 = “A complete absence of soreness,” 1 = “A light soreness in the muscle felt only when touched/a vague ache,” 2 = “A moderate soreness felt only when touched/a slight persistent pain,” 3 = “A light muscle soreness when walking up and down stairs,” 4 = “A light muscle soreness when walking on flat surface,” 5 = “A moderate muscle soreness, stiffness, or weakness when walking,” 6 = “A severe muscle soreness, stiffness, or weakness that limits my ability to move.” This scale has been used in previous studies investigating muscle soreness in the hamstring muscles (Gibson et al., 2006).

#### 2.5.3 Pain area

Pain area was assessed using a body chart on which participants were asked to mark all areas of pain. These charts were later digitized and imported into raster graphics software (Photoshop 6.0; Adobe, San Jose, CA, USA) where the selected areas were isolated and a pixel count determined (Cavaleri et al., 2019).

### 2.6 Functional measures

#### 2.6.1 Lower extremity functional scale

The LEFS was used to assess the functional status of the lower limb. The scale presents a series of everyday activities requiring the lower limb, with the participant recording a score from 4 (“no difficulty”) to 0 (“extreme difficulty or unable to perform”) for each item. The final score is summed, and a higher score (maximum of 80) represents lower levels of disability and greater function. The LEFS scale has excellent test–retest reliability (ICC 0.85–0.99) (Mehta et al., 2016).

#### 2.6.2 Maximum voluntary isometric contraction

The MVIC was measured using a Biodex isokinetic dynamometer (Biodex Multi‐Joint System 3, Shirley, NY, USA). Participants were seated upright, with their knees and hips in 90° of flexion. The participants were required to do a set of three maximal isometric knee flexions for 5 s each with 30 s rest in between each contraction (Dover et al., 2012). The highest measurement (in N m) was used for analysis.

#### 2.6.3 Single‐leg hop test

The horizontal single‐leg hop test provides insight into hamstring muscle function and injury risk (van der Harst et al., 2007). All participants were asked to perform the test barefoot, hopping forward as far as possible with the right leg. Hallux–hallux distance (cm) was recorded using a metric tape, and the greatest distance of three attempts was recorded.

### 2.7 Corticomotor organization

Corticomotor organization was assessed using TMS mapping. This technique is a noninvasive means by which to investigate the size, location, and excitability of corticomotor representations (Cavaleri et al., 2015). During TMS, electromyography (EMG) is used to record the muscle (biceps femoris) responses evoked following motor cortex stimulation. All mapping procedures were reported in accordance with the TMS‐specific methodological assessment checklist (Chipchase et al., 2012).

#### 2.7.1 Electromyography

Surface EMG (Ag–AgCl, Noraxon dual electrodes, interelectrode distance 2.0 cm) was used to record motor evoked potentials (MEPs) following TMS over the biceps femoris corticomotor representation. The active electrode was placed over the belly of the right biceps femoris muscle, and the ground electrode was positioned over the anterior superior iliac spine. EMG signals were amplified (×1000), bandpass filtered (20–1000 Hz), and sampled at 2 kHz using a Power 1401 Data Acquisition System and Signal3 software (Cambridge Electronic Design, Cambridge, United Kingdom).

#### 2.7.2 Hotspot and active motor threshold determination

During TMS, single‐pulse, biphasic stimuli were delivered using a Magstim Super Rapid^2^ Plus^1^ (Magstim Co. Ltd, Dyfed, UK) and a double 70 mm air‐cooled figure‐of‐eight coil. The coil was placed tangentially to the skull with the handle pointing posteriorly, inducing a current in the posterior–anterior direction (Richter et al., 2013). Visual feedback of EMG activity was provided via a monitor positioned at eye level 2 m in front of the participant.

The “hotspot” was defined as the coil position that evoked a maximal peak‐to‐peak MEP in the target muscle at a given stimulation intensity (Cavaleri et al., 2017a, 2017b; Ferreri & Rossini, 2013). The active motor threshold (aMT) was defined as the minimum TMS intensity required to elicit at least 5 discernible MEPs in a train of 10 stimuli delivered to the hotspot during a submaximal hamstring contraction (Groppa et al., 2012). A Brainsight stereotactic frameless neuronavigation system (Rogue Research Inc.) was used to determine the hotspot and aMT.

#### 2.7.3 TMS mapping protocol

During mapping, all stimuli were delivered, whereas the participant maintained a submaximal contraction of the hamstring muscles (10% of maximal EMG recorded during voluntary isometric knee flexion). The stimulation intensity was set at 110% of the aMT identified at baseline. Ninety stimuli were delivered pseudorandomly to the scalp over a 5 × 7 cm grid (six rows and eight columns) oriented to the cranial vertex (Cavaleri et al., 2018; Van De Ruit et al., 2015). The grid was superimposed on a generic brain image in the neuronavigation display. Using this system, each participant's cranial landmarks (nasion, inion, tragi, eye canthi, and cranial vertex) were registered at baseline and saved for use across sessions to ensure consistent grid placement, neuronavigation, and targeting.

#### 2.7.4 TMS map processing

Maps were generated offline using MATLAB using an approach adapted from previous studies (Cavaleri, 2022; Cavaleri et al., 2018; Van De Ruit et al., 2015). Briefly, triangular linear interpolation was used to create a full surface map within a transformed plane containing stimulation coordinates and their corresponding MEP amplitudes. The resultant map was divided into 2500 partitions (50 × 50). Partitions with MEP amplitudes exceeding 25% of a participant's peak response were labeled as “active.” To calculate map area, the number of active partitions was divided by 2500 and multiplied by the size of the stimulated area. Map volume was determined by summing the approximated MEP amplitudes (in millivolts) of all active partitions in the matrix. The center of gravity (CoG), or amplitude weighted center of the map, for each muscle was calculated using the formula: CoG = Σ(*xz*)/Σ*z*; Σ(*yz*)/Σ*z* (where *x* = mediolateral coordinate; *y* = anteroposterior coordinate; and *z* = corresponding MEP amplitude). Shifts in the location of the CoG were calculated as the Euclidean distance (ED) from baseline using the formula: ED = √(*y*1 − *y*2)^2^ + (*x*1 − *x*2)^2^, (where *y* = anteroposterior coordinate; *x* = mediolateral coordinate; and 1 and 2 refer to baseline and post‐training values, respectively) (Cavaleri et al., 2018; Van De Ruit et al., 2015).

### 2.8 Statistical analyses

All statistical analyses were performed using the Statistical Package for the Social Sciences software (version 23; IBM Corp, Armonk, NY, USA). Statistical significance was set at *p* < .05.

#### 2.8.1 Effect of experimental hamstring pain on mechanical sensitivity, pain, function, and corticomotor outcomes

The effect of sustained experimental hamstring pain on mechanical sensitivity (PPTs), pain (numerical rating scale, muscle soreness, pain area), functional (LEFS, MVICs, single‐leg hop test), and corticomotor (map volume, map area, CoG displacement) outcomes was analyzed using mixed‐model ANOVAs with factors “group” (experimental vs. control) and “time” (Baseline and Day 2 postexercise). The Shapiro–Wilk test and Mauchly's test of sphericity were applied to assess assumption of normality and sphericity, respectively (Moulton, 2010; Shapiro & Wilk, 1965). The Greenhouse–Geisser correction for non‐sphericity was applied for datasets that violated the assumption of sphericity (Abdi, 2010). Where appropriate, post hoc analyses were performed by using Sidak‐adjusted multiple comparison tests.

#### 2.8.2 Variability in corticomotor responses to experimental hamstring pain

Previous studies have found that corticomotor reorganization in response to pain has high interindividual variability, with people displaying either a decrease in corticomotor excitability (“depression”) or an increase (“facilitation”) that could affect pain experiences (Cavaleri et al., 2021; Seminowicz et al., 2019). Participants in the experimental group were therefore categorized as “depressors” or “facilitators” in terms of both their map volume and map area changes during hamstring pain. To do so, the mean absolute % changes in map volume (Δvolume) and map area (Δarea) from baseline in the control group were determined. Participants in the experimental group were classified as facilitators if, at Day 2 postexercise, their mean % change in map volume or map area from baseline exceeded the Δvolume or Δarea in the control group by greater than 1 standard deviation (SD) (Cavaleri et al., 2020). Conversely, participants were classified as depressors if their change in map volume or area was at least 1 SD below the Δvolume or Δarea in the control group. All other participants were classified as nonresponders.

#### 2.8.3 Relationship among corticomotor reorganization, pain, and function

To determine whether facilitators in the experimental group exhibited different mechanical sensitivity, pain, and function changes to depressors, repeated‐measures ANOVAs were conducted with the between‐subject factor “Response” (facilitators vs. depressors) and the within‐subject factor “time” (Baseline and Day 2 postexercise). Nonresponders were excluded from this analysis. Pearson's correlations were run to further explore the relationship between changes in corticomotor outcomes and changes in mechanical sensitivity, pain, and function. The following values were used to interpret Pearson's correlation coefficient: *r* < 0.3 = weak correlation; *r* between 0.3 and 0.5 = moderate correlation; and *r* > 0.5 = strong correlation (Laerd Statistics, 2020).

## 3 RESULTS

### 3.1 Participant characteristics

Participant characteristics are summarized in Table 1. The experimental (eccentric) and control (concentric) groups were comparable on all baseline variables. All participants attended the sessions as intended and no adverse events were reported. There was no significant difference in RPE between groups over the course of the exercise protocols (time: *F*_3.5, 119.1_ = 40.06, *p* < .01; group: *F*_1, 34_ = 0.01, *p* = .94; group × time: *F*_3.5, 119.1_ = 1.05, *p* = .38).

**TABLE 1:** Participant characteristics.

	Experimental group mean (SD)	Control group mean (SD)	*p* Value
Sample size (*n*)	26	10	–
Sex (male, female)	11, 15	5, 5	–
Age (years)	22 (4)	23 (4)	.58
Height (cm)	168.5 (9.6)	169.9 (6.6)	.67
Weight (kg)	72.3 (19.6)	70.8 (12.3)	.83
Baseline aMT (%)	85 (13)	78 (17)	.18
DASS‐21 depression	1.9 (2.6)	2.3 (3.6)	.73
DASS‐21 anxiety	2.0 (2.1)	2.1 (2.1)	.90
DASS‐21 stress	3.0 (3.0)	2.1 (3.0)	.41

Abbreviations: aMT, active motor threshold; cm, centimeters; DASS‐21, depression anxiety stress scale; kg, kilograms; *n*, number; SD, standard deviation.

#### 3.1.1 Effect of experimental hamstring pain on mechanical sensitivity, pain, and function

There was no significant difference between the experimental (pain‐inducing eccentric exercise) and control (pain‐free concentric exercise) groups in terms of mechanical sensitivity, assessed using PPTs (time: *F*_1, 34_ = 0.01, *p* = .78; group: *F*_1, 34_ = 1.76, *p* = .91; group × time: *F*_1, 34_ = 2.41, *p* = .13). However, the experimental group had greater pain at rest (*p* < .01), when reaching for the floor with knees straight (*p* < .01), and during the hamstring‐drag test (*p* < .01) compared to the control group on Day 2 (Table 2). As shown in Table 2, the experimental group also reported greater muscle soreness (*p* < .01) and a larger pain area (*p* < .01) than the control group.

**TABLE 2:** Differences in pain outcomes between groups.

	Mean (SD) values on Day 2					
Outcomes	Eccentric	Concentric	*t*	df	MD (95% CI)	*p* Value
Pain (NRS)						
‐At rest	1.3 (1.2)	.0 (.0)	3.37	34	1.3 (0.5–2.1)	<.01*
Reaching for floor	5.4 (2.5)	.2 (.4)	6.55	34	5.2 (3.6–6.8)	<.01*
Hamstring‐drag test	3.4 (2.1)	.0 (.0)	4.87	34	3.4 (2.0–4.8)	<.01*
Muscle soreness	4.5 (1.8)	.2 (.4)	7.20	34	4.3 (3.1–5.5)	<.01*
Pain area	25.9 (16.6)	.2 (.4)	4.85	34	25.7 (14.9–36.4)	<.01*

Abbreviations: CI, confidence interval; df, degrees of freedom; MD, mean difference; NRS, numerical rating scale; SD, standard deviation.

*p < .05.

There were also differences between groups in terms of function following the exercise protocol. A significant group × time interaction was observed for LEFS scores (time: *F*_1, 34_ = 29.72, *p* < .01; group: *F*_1, 34_ = 20.93, *p* < .01; group × time: *F*_1, 34_ = 20.93, *p* < .01). Post hoc analyses revealed that LEFS scores in the experimental group (51.4 ± 17.5) were lower than those of the control group (77.5 ± 5.9) on Day 2, reflecting poorer lower limb function (*p* < .01). There were also differences between groups in MVIC recordings over time (time: *F*_1, 34_ = 11.58, *p* < .01; group: *F*_1, 34_ = 2.67, *p* = .11; group × time: *F*_1, 34_ = 25.87, *p* < .01). Specifically, the experimental group had lower MVIC recordings (60.8 ± 41.2) than the control group (103.2 ± 41.2) (*p* < .01) on Day 2. However, there was no difference between groups in single‐leg hop performance (time: *F*_1, 34_ = 0.18, *p* = .68; group: *F*_1, 34_ = 1.31, *p* = .26; group × time: *F*_1, 34_ = 1.13, *p* = .72).

#### 3.1.2 Effect of experimental hamstring pain on corticomotor organization

**FIGURE 1:**
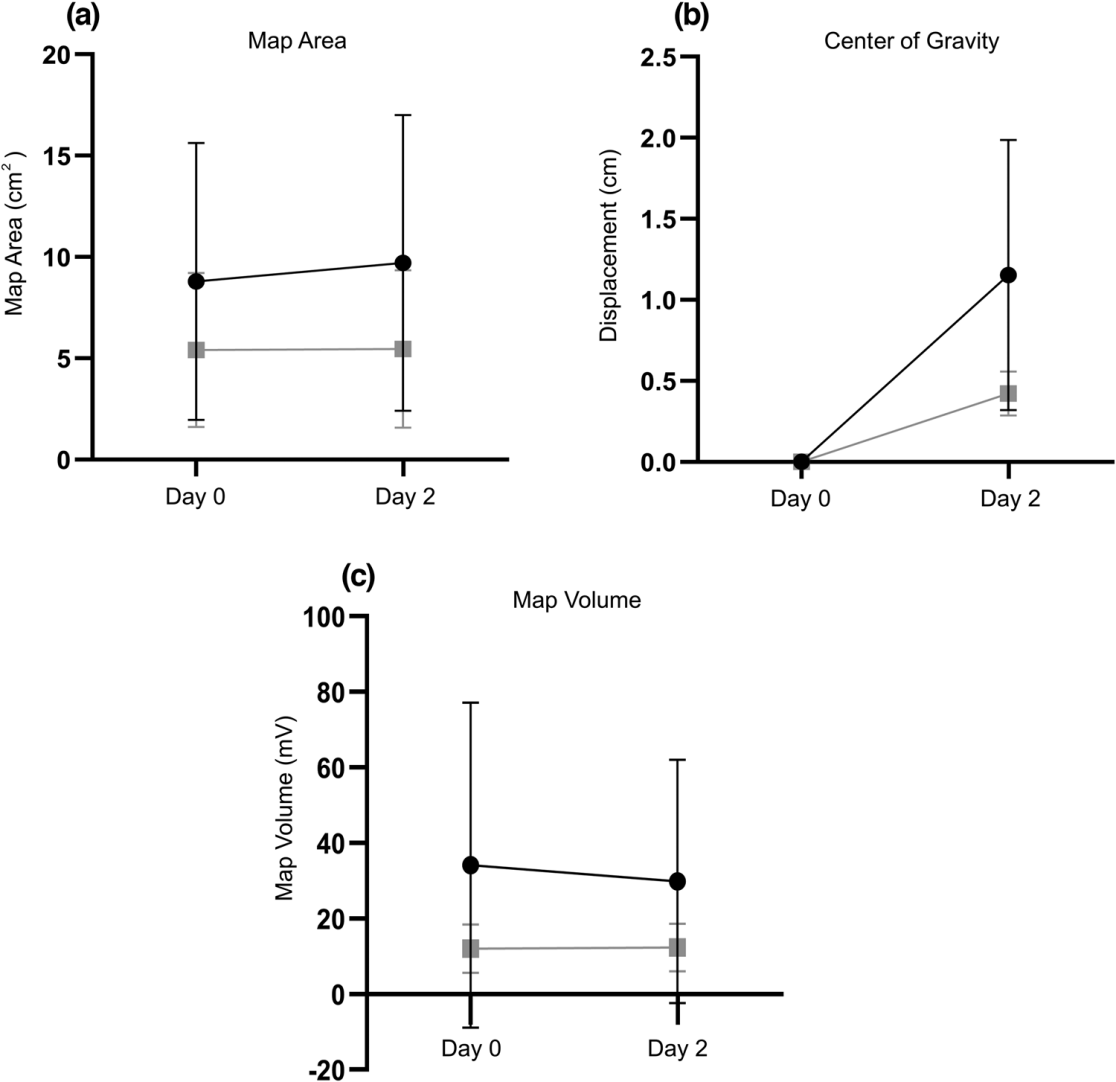
**Changes in transcranial magnetic stimulation (TMS) mapping outcomes (A: Map Area, B: Centre of Gravity, C: Map Volume) over time**. Gray line: control group, black line: experimental group, cm: centimeters, mV: millivolts.

There were no overall changes in map area (time: *F*_1, 34_ = 0.18, *p* = .68; group: *F*_1, 34_ = 3.35, *p* = .08; group × time: *F*_1, 34_ = 0.14, *p* = .71) or volume (time: *F*_1, 34_ = 0.10, *p* = .76; group: *F*_1, 34_ = 3.68, *p* = .06; group × time: *F*_1, 34_ = 0.13, *p* = .72) in either the experimental or control groups, suggesting no predictable change in corticomotor excitability in response to acute experimental hamstring pain. However, there were significant differences in CoG displacement, reflective of corticomotor reorganization, between groups (*t*_34_ = 2.73, *p* = .01). Specifically, the experimental group demonstrated greater CoG displacement from baseline (1.2 ± 0.8) than the control group (0.4 ± 0.1). Figure 1 presents a summary of the changes in TMS mapping outcomes over time for each group.

### 3.2 Variability in corticomotor responses to experimental hamstring pain

Changes to the representation of biceps femoris in response to sustained experimental pain were variable. Indeed, the mean absolute Δvolume ± SD in the experimental group was 177% ± 364%, and the mean absolute Δarea ± SD was 87% ± 130%. Conversely, the control group exhibited little variability in corticomotor responses over time. The mean absolute Δvolume ± SD in the control group was 9% ± 3%, and the Δarea + SD in this group was 12% ± 7%.

Based upon the criteria outlined in Section 2, 12 (46%) participants in the experimental group were classified as facilitators, 12 (46%) as depressors, and 2 (8%) as nonresponders in terms of map volume. In terms of map area, 10 (39%) participants in the experimental group were classified as facilitators, 11 (42%) as depressors, and 5 (19%) as nonresponders. Center of gravity displacement ranged from 0 to 3 cm. All participants in the control group were considered to be “nonresponders.”

### 3.3 Relationship among corticomotor reorganization, pain, and function

As shown in Table 3, a strong inverse correlation between map volume and PPTs was identified.

**TABLE 3:** Correlations among corticomotor reorganization, pain, and function.

	Correlation coefficient (*p* value)								
	PPT	Pain at rest (NRS)	Reaching for floor (NRS)	Hamstring drag (NRS)	Pain area	Muscle soreness	LEFS	MVC	SLH
Map area	−.46 (.02)*	.05 (.79)	.06 (.77)	.16 (.42)	.25 (.22)	.14 (.49)	−.16 (.45)	−.02 (.94)	−.31 (.12)
Map vol.	−.56 (<.01)*	.07 (.72)	.21 (.30)	.14 (.48)	.22 (.28)	.17 (.40)	−.20 (.32)	−.05 (.80)	−.17 (.40)
CoG disp.	.22 (.29)	−.16 (.44)	−.36 (.07)	−.04 (.86)	−.08 (.71)	−.32 (.11)	−.04 (.84)	−.28 (.17)	−.03 (.88)

Abbreviations: CoG disp., center of gravity displacement; LEFS, lower extremity functional scale; MVIC, maximal voluntary isometric contraction; NRS, numeric rating scale; PPT, pressure pain threshold; SLH, single‐leg hop.

*p < .05.

**FIGURE 2:**
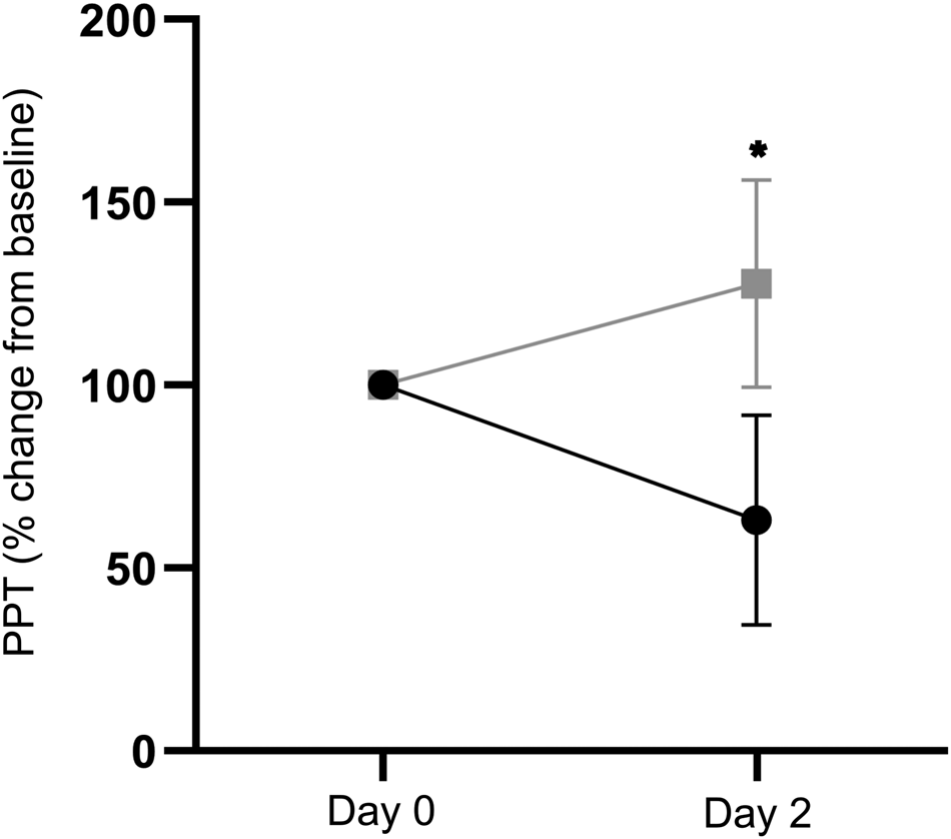
Difference in pressure pain thresholds (PPTs) between facilitators and depressors. Gray line: depressors (map volume), black line: facilitators (map volume), *p < .05.

There was a significant map volume response × time interaction for PPTs (time: *F*_1, 22_ = 2.73, *p* = .11; group: *F*_1, 22_ = 0.33, *p* = .57; group × time: *F*_1, 24_ = 7.33, *p* = 0 < .01). Post hoc analyses revealed that individuals who demonstrated increases in map volume (corticomotor facilitation) had significantly lower PPTs (increased mechanical sensitivity) on Day 2 than those demonstrating reductions in map volume (corticomotor depression) (*p* = .04) (Figure 2). Similarly, increases in map area during experimental hamstring pain were moderately‐to‐strongly correlated with reductions in PPTs. A repeated measures ANOVA revealed a significant map area response × time interaction for PPTs (time: *F*_1, 19_ = 2.87, *p* = .11; group: *F*_1, 19_ = 0.48, *p* = .50; group × time: *F*_1, 19_ = 13.95, *p* < .01), with individuals demonstrating increases in map area possessing lower PPTs on Day 2 than those demonstrating reductions in map area (*p* = .04). As shown in Table 3, no further correlations between corticomotor outcomes and pain or function were identified.

## 4 DISCUSSION

This was the first study to explore the variability of corticomotor reorganization in response to sustained lower limb pain. Corticomotor responses were variable, with 46% of participants displaying corticomotor facilitation, 46% displaying corticomotor depression, and 8% being considered nonresponders based upon map volume. Further investigation of this variability revealed that corticomotor facilitation was strongly correlated with greater mechanical sensitivity (lower PPTs). Corticomotor depression was correlated with lower mechanical sensitivity (higher PPTs), suggesting a potentially protective response. Corticomotor reorganization was not associated with changes in pain intensity (numerical rating scale) or function. These data provide novel insights into the potential mechanisms underlying recurrent hamstring pain and injury.

The variable corticomotor changes observed in response to sustained hamstring pain are consistent with those reported in studies of the upper limb. A recent systematic review and meta‐analysis of 90 data points across five studies demonstrated that, relative to baseline, 57% of data points taken during sustained pain reflected decreased corticomotor excitability and 43% reflected increased excitability. Such variability in corticomotor responses is congruent with that observed following both motor skill learning (Cavaleri et al., 2020; van de Ruit & Grey, 2019) and noninvasive brain stimulation (Fratello et al., 2006; Martin et al., 2006; Müller‐Dahlhaus et al., 2008; Wiethoff et al., 2014). Though yet to be completely elucidated, this variability is thought to be driven by interindividual differences in factors such as anatomy (e.g., cortical thickness) (Ridding & Ziemann, 2010), kinesiophobia (Summers et al., 2020), and history of exercise (Ridding & Ziemann, 2010). Prior synaptic activity in a cortical region is also thought to influence subsequent reorganization. For example, Siebner et al. (2004) found that corticomotor responses to repetitive TMS can be manipulated by prior administration of either anodal or cathodal transcranial direct current stimulation. It is therefore plausible that an individual's history of pain and associated synaptic adaptations could influence their corticomotor responses to subsequent pain experiences.

The relationships between corticomotor outcomes and mechanical sensitivity observed in the present study contrast with that observed in studies of sustained upper limb pain. Previous work has demonstrated that corticomotor depression is associated with reduced upper limb pain severity during pain lasting minutes‐to‐hours but increased pain severity during sustained pain (lasting days‐to‐weeks) (Chowdhury et al., 2022). Consistent with existing hypotheses (Hodges & Tucker, 2011), these studies suggest that corticomotor depression may be a beneficial short‐term adaptation to pain, but persistence of this adaptation may be associated with poorer long‐term outcomes. However, previous work examining corticomotor responses to sustained pain has been limited to the upper limb, with a recent systematic review acknowledging that region‐specific corticomotor responses have yet to be adequately explored (Chowdhury et al., 2022).

The present study extends existing literature by demonstrating that, in contrast to findings from upper limb studies, corticomotor depression may represent a beneficial response during sustained hamstring pain. However, it is important to note that, although corticomotor depression was associated with improved mechanical sensitivity, no such relationship was observed in terms of pain intensity (on a numerical rating scale). The reason for this discrepancy is unclear, but it should also be noted that PPTs reflect a distinct mechanism in peripheral neural sensitivity, whereas pain intensity is a subjective report that may encapsulate a greater degree of psychosocial influences. Indeed, previous work has demonstrated that pain intensity is not necessarily correlated with areas of increased mechanical sensitivity in the lower limb (Sánchez Romero et al., 2020).

It is reasonable to postulate that corticomotor adaptations to pain could be region‐specific. The upper limb is responsible for fine motor tasks, so a reduction in corticomotor excitability during acute pain in this region is thought to be beneficial as a means of restricting movement and ensuring protection from further harm (Cavaleri et al., 2021; Hodges & Tucker, 2011). In contrast, the lower limb is crucial to locomotion, so an increase in corticomotor excitability could plausibly facilitate escape from harm (Hodges & Tucker, 2011). Consistent with contemporary theories of motor adaptation to pain (Hodges & Tucker, 2011), persistence of these early corticomotor responses could elicit detrimental long‐term consequences. Indeed, persistent increases in quadriceps corticomotor excitability have been observed in individuals with patellar tendinopathy (Rio et al., 2016) compared to healthy controls. The relationship between increased sensitivity and corticomotor facilitation observed in this study supports that, while potentially protective in the upper limb (Chowdhury et al., 2022), ongoing increases in corticomotor excitability may contribute toward poorer outcomes in lower limb pain or injury. However, further work exploring the transition from acute to sustained and chronic pain is required to confirm these hypotheses.

### 4.1 Future directions and clinical implications

The results of this study are of potential clinical importance. Increasing emphasis has been placed upon the need for prognostic indicators of lower limb recovery or pain persistence (Sánchez Romero et al., 2021). Our data raise the possibility that corticomotor facilitation in response to hamstring pain could indicate susceptibility to greater mechanical sensitivity and contribute to delayed return‐to‐play following hamstring pain or injury. Indeed, pain on palpation (an index of mechanical sensitivity) is used commonly to progress hamstring rehabilitation, with higher pain sensitivity predictive of longer return‐to‐play times after hamstring injury (Hickey et al., 2022). Higher mechanical sensitivity may also impede engagement with the rehabilitation process, potentially impacting hamstring recovery and reinjury risk. As such, techniques aimed at restoring corticomotor excitability, and therefore mechanical sensitivity, may aid the rehabilitation process following a hamstring injury. Indeed, the use of noninvasive brain stimulation has shown promise as a means of expediting recovery of experimental and clinical musculoskeletal pain (Borovskis et al., 2021; Cavaleri et al., 2019; Moisset et al., 2016; Moukhaiber et al., 2022; O'Connell et al., 2018) and mechanical sensitivity has been suggested as a potential marker for evaluating the success of such strategies (Pedersini et al., 2020). However, given that numerical ratings of pain and function in the present study were not impacted by changes in corticomotor excitability, these clinical implications remain speculative. Larger datasets, in a hamstring‐injured cohort, are required to confirm the current findings and understand the impact of corticomotor reorganization on injury prognosis and risk of reinjury.

This study has several strengths. A control group (concentric exercise) was employed, mitigating the potential confounding effects of repetition and fatigue on corticomotor outcomes. Assessors were also blinded to minimize measurement bias. However, this study is not without limitations. The results of this study are based on an experimental pain model (DOMS) that mimics signs and symptoms associated with hamstring injury (pain on palpation and loss of function). Whether our results can be translated to clinical hamstring pain requires further investigation. Another potential limitation of this study is that outcome measures were not followed‐up beyond the resolution of pain. Previous studies have shown maintenance of corticomotor adaptations following experimentally‐induced pain in the upper limb (Burns et al., 2016; Chowdhury et al., 2022). Exploring whether a similar phenomenon occurs in response to hamstring pain would have important implications for rehabilitation and long‐term reinjury risk.

Finally, it is important to acknowledge that the reliability of TMS mapping requires further exploration. Although the technique employed has demonstrated excellent within‐ and between‐session reliability when assessing corticomotor representations of upper limb (Cavaleri et al., 2018, 2019, 2021; Van De Ruit et al., 2015) and low back (Cavaleri et al., 2020) musculature, the reliability of rapid TMS mapping involving the lower limb is yet to be completely elucidated. Upper limb muscles possess large, relatively superficial and excitable corticomotor representations, meaning that they readily elicit consistent MEPs (Palmer & Ashby, 1992; Wassermann et al., 1992). Conversely, lower limb muscles have fewer corticospinal projections arising from a relatively small and deep cortical area, making it more difficult to obtain stable MEPs (Palmer & Ashby, 1992; Wassermann et al., 1992). Lower limb muscles also have a greater proportion of ipsilateral projections from M1 than upper limb muscles (Strutton et al., 2004). The reliability of map recordings may therefore differ between these regions. Accordingly, it is plausible that a degree of the variability observed throughout this study was attributable to methodological factors. However, given the minimal variability observed over time in the control group (mean absolute Δvolume < 10% in the control group vs. Δvolume > 175% in the experimental group), it is valid to suggest that most of the variability observed in the experimental group was attributable to pain rather than being a product of methodological factors or time.

## 5 CONCLUSION

This study is the first to explore the variability of corticomotor responses to experimentally‐induced sustained hamstring pain. In contrast to findings from the upper limb, individuals who developed corticomotor facilitation in response to hamstring pain experienced greater mechanical sensitivity than those who developed corticomotor depression. These novel data could have implications for rehabilitation following hamstring pain or injury. Further work in clinical populations is required to determine whether interindividual changes in corticomotor outcomes impact prognosis and risk of reinjury.

## AUTHOR CONTRIBUTIONS

Rocco Cavaleri, Simon J. Summers, and Ebonie Rio were all involved in the design, writing, and editing of the study and manuscript. Rocco Cavaleri, Syed Jawwad Imam, Nadia Moukhaiber, Daniel Thomson, and Ariane Suhood were all involved in data collection. The final manuscript was approved by all authors.

## CONFLICT OF INTEREST STATEMENT

Dr Ebonie Rio, a senior research fellow, NHMRC, was funded as an early career researcher. There are no conflict of interests or additional acknowledgments to report.

### PEER REVIEW

The peer review history for this article is available at https://publons.com/publon/10.1002/brb3.2996.

## Data Availability

Individual‐level data that support the findings of this study are available from the corresponding author, Rocco Cavaleri, upon reasonable request.
